# Supplementary material for: N-Acetylchitooligosaccharides Alleviate Pulmonary Inflammation and Modulate Glycerophospholipid Metabolism in Murine Acute Lung Injury
Source: Int J Mol Sci. 2025 Sep 18;26(18):9128. doi: 10.3390/ijms26189128 (PMC12470115; doi:10.3390/ijms26189128)
Supplement: Supplementary file 1 [file ijms-26-09128-s001.zip › ijms-3808091-supplementary.pdf]

Supplementary data

Supplementary Tables

Table S1. List of the differentiated metabolites in plasma.

NO.	Ion Form	m/z(Da)	Compound name	HMDB	VIP	P value	Fold change
1	[M+H]+	508.35221	Daphniphylline	HMDB0030291	2.37014	4.14E-06	0.18572
2	[M+H]+	569.38071	Lansioside C	HMDB0035103	2.36562	4.57E-06	0.20031
3	[M+H]+	509.35506	3alpha,7alpha,12alpha,24(S)-tetrahydroxy-5beta-cholestan-27-al	HMDB0062206	2.35633	5.56E-06	0.16946
4	[M+H]+	571.38231	PA(14:1(9Z)/14:0)	HMDB0114795	2.29216	1.73E-05	0.18510
5	[M+H]+	337.05621	5-Aminoimidazole-4-carboxamide-1-β-d-ribofuranosyl 5-monophosphate	-	2.28979	6.20E-06	2.17190
6	[M+H]+	269.23753	14-Methylhexadecanoic Acid	HMDB0031067	2.26421	7.31E-05	0.28971
7	[M+H]+	441.12207	Enterodiol sulfate	HMDB0240504	2.24421	1.76E-05	4.42480
8	[M+H]+	308.26695	Dicyclomine	HMDB0014942	2.16268	5.17E-05	0.30994
9	[M+H]+	347.16594	Arginyl-Glutamine	HMDB0028707	2.13631	4.58E-04	0.09214
10	[M+H]+	341.29894	Polyoxyethylene (600) monoricinoleate	HMDB0032476	2.12398	1.36E-04	0.46037
11	[M+H]+	181.16123	Allyl nonanoate	HMDB0029763	2.11705	4.89E-04	0.18029
12	[M+H]+	233.06046	7-Hydroxy-2-methylisoflavone	HMDB0033979	2.07814	3.03E-04	2.50740
13	[M+H]+	278.05716	Cidofovir	HMDB0014513	2.07013	3.57E-04	2.18870
14	[M+H]+	266.26267	Avocadenofuran	HMDB0030932	2.06678	4.85E-04	0.34834
15	[M+H]+	297.28732	Methyl oleate	-	2.06148	5.35E-03	0.17718
16	[M+H]+	626.48970	Galactosylceramide (d18:1/12:0)	HMDB0012320	2.05965	3.20E-04	0.48645

**17**	**[M+H]+**	**265.25920**	**Hexadecan-1-ol**	**HMDB0003424**	**2.05765**	**5.45E-04**	**0.34552**
**18**	**[M+H]+**	**134.03683**	**Pyrrole-2-carboxylic acid**	**HMDB0004230**	**2.05168**	**2.35E-04**	**2.65480**
**19**	**[M+H]+**	**340.29572**	**Oleoyl glycine**	**HMDB0013631**	**2.04992**	**4.79E-04**	**0.37269**
**20**	**[M+H]+**	**196.03704**	**Glycerylphosphorylethanolamine**	**HMDB0000114**	**2.04091**	**1.52E-03**	**2.12240**
**21**	**[M+H]+**	**307.27223**	**5,8,11-Eicosatrienoic acid**	**HMDB0010378**	**2.04024**	**6.54E-04**	**0.18871**
**22**	**[M+H]+**	**188.98263**	**Hydroquinone sulfate**	**HMDB0240263**	**2.03590**	**2.82E-04**	**2.11750**
**23**	**[M+H]+**	**585.36958**	**3-Epipapyriferic acid**	**HMDB0040498**	**2.01108**	**6.58E-04**	**0.09511**
**24**	**[M+H]+**	**324.26519**	**trans-2-Dodecenoylcarnitine**	**HMDB0013326**	**2.00917**	**8.80E-04**	**0.39833**
**25**	**[M+H]+**	**307.26318**	**Cis-11,14-Eicosadienoic acid**	**HMDB0005060**	**2.00471**	**4.49E-04**	**0.37365**
**26**	**[M+H]+**	**587.23562**	**Dutasteride**	**HMDB0015258**	**1.98945**	**1.54E-05**	**0.41957**
**27**	**[M+H]+**	**238.22988**	**Solamine**	**HMDB0031202**	**1.98036**	**1.78E-03**	**0.25669**
**28**	**[M+H]+**	**166.07020**	**Carbazole**	**-**	**1.97062**	**9.07E-04**	**2.28940**
**29**	**[M+H]+**	**267.07985**	**Inosine**	**HMDB0000195**	**1.95616**	**1.47E-03**	**2.22960**
**30**	**[M+H]+**	**237.22648**	**Ambroxane**	**HMDB0036831**	**1.94823**	**1.98E-03**	**0.28425**
**31**	**[M+H]+**	**255.23807**	**Palmitoleic acid**	**HMDB0003229**	**1.94528**	**2.00E-03**	**0.30071**
**32**	**[M+H]+**	**380.06806**	**Celecoxib**	**HMDB0005014**	**1.94299**	**1.00E-02**	**2.95530**
**33**	**[M+H]+**	**220.21838**	**Acetaldehyde diisoamyl acetal**	**HMDB0032153**	**1.93920**	**2.03E-03**	**0.13102**
**34**	**[M+H]+**	**223.16405**	**L-Menthyl (R,S)-3-hydroxybutyrate**	**HMDB0032370**	**1.93115**	**1.48E-03**	**0.32008**
**35**	**[M+H]+**	**286.07789**	**Clitocine**	**HMDB0033718**	**1.91871**	**7.23E-04**	**2.26700**
**36**	**[M+H]+**	**326.09350**	**p-Acetamidophenyl-β-D-glucuronide**	**HMDB0010316**	**1.90376**	**9.81E-03**	**2.01400**
**37**	**[M+H]+**	**343.12701**	**Maltitol**	**HMDB0002928**	**1.82724**	**2.98E-05**	**5.07860**
**38**	**[M+H]+**	**365.25917**	**6-O-Acetylaustroinulin**	**HMDB0036803**	**1.81698**	**2.84E-04**	**0.44677**
**39**	**[M+H]+**	**343.27435**	**4,7,10,13,16,19-Docosahexaenoic acid methyl ester**	**-**	**1.78079**	**6.90E-04**	**0.42772**
**40**	**[M+H]+**	**440.07522**	**dehydrofelodipine**	**HMDB0061029**	**1.76765**	**1.21E-02**	**2.01350**
**41**	**[M+H]+**	**344.27770**	**Avocadyne 2-acetate**	**HMDB0031047**	**1.70993**	**1.44E-03**	**0.46013**
**42**	**[M+H]+**	**320.27622**	**MG(0:0/14:0/0:0)**	**HMDB0011530**	**1.65192**	**5.49E-04**	**3.01460**

**43**	**[M+H]+**	**269.25416**	**Metyl palmitoleate**	**-**	**1.64530**	**8.32E-07**	**6.05330**
**44**	**[M+H]+**	**319.27252**	**cannitine C10:0-d3**	**-**	**1.63614**	**7.55E-04**	**0.49636**
**45**	**[M+H]+**	**309.10570**	**Thiamine**	**HMDB0000235**	**1.62532**	**9.41E-03**	**2.00740**
**46**	**[M+H]+**	**258.25717**	**Decanoylcholine**	**HMDB0013228**	**1.61869**	**9.39E-03**	**0.48285**
**47**	**[M+H]+**	**236.03904**	**N-Acetylhistidine**	**HMDB0032055**	**1.58455**	**5.87E-04**	**0.41887**
**48**	**[M+H]+**	**241.11582**	**γ-Glu-Leu**	**HMDB0011171**	**1.55104**	**9.34E-03**	**2.13950**
**49**	**[M+H]+**	**242.11874**	**Agomelatine**	**HMDB0015636**	**1.53994**	**9.06E-03**	**2.12010**
**50**	**[M+H]+**	**225.12793**	**2-Amino-1methyl-6-phenylimidazo[4,5-b]pyridine**	**HMDB0041008**	**1.46703**	**1.50E-03**	**0.46742**
**51**	**[M+H]+**	**157.05979**	**L-β-Imidazolelactic acid**	**-**	**1.42147**	**1.32E-03**	**0.46667**
**52**	**[M+H]+**	**756.59524**	**PE-NMe2(18:0/18:1(9Z))**	**HMDB0113871**	**1.42047**	**2.50E-10**	**2.29740**
**53**	**[M+H]+**	**739.38559**	**Glucoconvallasaponin B**	**HMDB0034327**	**1.39440**	**1.89E-03**	**0.33122**
**54**	**[M+H]+**	**382.09175**	**N-(1-Deoxy-1-fructosyl)tyrosine**	**HMDB0037845**	**1.33338**	**1.29E-03**	**0.45237**
**55**	**[M+H]+**	**199.12922**	**5-Methyl-2-(1-methylethyl)-cyclohexanone**	**HMDB0061802**	**1.29052**	**4.68E-05**	**0.43384**
**56**	**[M+H]+**	**143.10047**	**2-Propylpentanoic acid**	**HMDB0001877**	**1.27987**	**3.58E-06**	**0.44782**
**57**	**[M+H]+**	**289.09985**	**Fluconazole**	**HMDB0014342**	**1.24297**	**7.28E-05**	**2.30970**
**58**	**[M+H]+**	**393.22785**	**7';-Carboxy-gamma-chromanol**	**HMDB0012850**	**1.23715**	**3.79E-05**	**0.44661**
**59**	**[M+H]+**	**267.13849**	**Dienestrol**	**HMDB0015027**	**1.92249**	**2.11E-06**	**0.34622**
**60**	**[M+H]+**	**157.11500**	**N-Methylnicotinium**	**HMDB0001009**	**1.90044**	**3.52E-06**	**0.30847**
**61**	**[M+H]+**	**333.28609**	**3,4-Dimethyl-5-pentyl-2-furanundecanoic acid**	**HMDB0031126**	**1.88339**	**1.93E-06**	**5.02990**
**62**	**[M+H]+**	**421.33469**	**27-Nor-5b-cholestane-3a,7a,12a,24,25-pentol**	**HMDB0002126**	**1.85566**	**6.64E-06**	**0.16596**
**63**	**[M+H]+**	**422.33775**	**3-Epidemissidine**	**HMDB0032023**	**1.84305**	**9.00E-06**	**0.18832**
**64**	**[M+H]+**	**435.35283**	**Cholestane-3,7,12,24,25-pentol**	**HMDB0002208**	**1.78947**	**3.75E-05**	**0.13557**
**65**	**[M+H]+**	**585.27003**	**Absintholide**	**HMDB0038171**	**1.74005**	**8.17E-05**	**0.45895**
**66**	**[M+H]+**	**737.35496**	**Bismahanine**	**HMDB0040773**	**1.73796**	**2.54E-04**	**0.49934**
**67**	**[M+H]+**	**518.34647**	**Physalolactone B**	**HMDB0034200**	**1.66507**	**3.61E-04**	**2.63520**
**68**	**[M+H]+**	**437.24723**	**Doxapram**	**HMDB0014701**	**1.66148**	**2.60E-04**	**0.31180**

**69**	**[M+H]+**	**403.19662**	**D-Linalool 3-(6';';-malonylglucoside)**	**HMDB0040729**	**1.65901**	**2.51E-04**	**4.04150**
**70**	**[M+H]+**	**429.16041**	**b-D-Xylopyranosyl-(1->4)-a-L-rhamnopyranosyl-(1->2)-L-arabinose**	**HMDB0041221**	**1.65830**	**1.91E-04**	**7.63730**
**71**	**[M+H]+**	**457.17744**	**2-amino-4-({1-[(carboxymethyl)-C-hydroxycarbonimidoyl]-2-[(3-oxo-1-phenylpropyl)sulfanyl]ethyl}-C-hydroxycarbonimidoyl)butanoic acid**	**HMDB0135282**	**1.64565**	**3.00E-04**	**5.14510**
**72**	**[M+H]+**	**412.22339**	**Methysergide**	**HMDB0014392**	**1.64240**	**3.76E-04**	**0.22255**
**73**	**[M+H]+**	**463.38286**	**6-Deoxohomodolichosterone**	**HMDB0034430**	**1.64210**	**3.35E-04**	**0.26155**
**74**	**[M+H]+**	**510.28325**	**2-(2,4-dihydroxyphenyl)-3-(3,7-dimethylocta-2,6-dien-1-yl)-5,7-dihydroxy-6-(3-methylbut-2-en-1-yl)-3,4-dihydro-2H-1-benzopyran-4-one**	**HMDB0129721**	**1.62748**	**3.41E-04**	**7.25310**
**75**	**[M+H]+**	**279.05570**	**Sulfametopyrazine**	**HMDB0014802**	**1.62375**	**1.17E-03**	**2.10780**
**76**	**[M+H]+**	**341.16499**	**Gliclazide**	**HMDB0015252**	**1.62202**	**4.25E-04**	**4.75470**
**77**	**[M+H]+**	**413.18568**	**Cinalukast**	**HMDB0014725**	**1.62094**	**4.36E-04**	**8.80260**
**78**	**[M+H]+**	**805.43630**	**Madlongiside D**	**HMDB0037905**	**1.61979**	**5.22E-04**	**19.69900**
**79**	**[M+H]+**	**611.26437**	**Ganoderic acid H**	**HMDB0035987**	**1.61649**	**4.37E-04**	**15.98900**
**80**	**[M+H]+**	**597.37806**	**3';-N-Acetyl-4';-O-(10,12-octadecadienoyl)fusarochromanone**	**HMDB0038566**	**1.61396**	**4.78E-04**	**12.52000**
**81**	**[M+H]+**	**405.22525**	**Hydrocortisone 21-acetate**	**HMDB0000063**	**1.61395**	**4.17E-04**	**29.75000**
**82**	**[M+H]+**	**410.22597**	**Sphingosine 1-phosphate (d16:1-P)**	**HMDB0060061**	**1.61374**	**5.49E-04**	**0.22083**
**83**	**[M+H]+**	**488.14366**	**Bacampicillin**	**HMDB0015540**	**1.61206**	**4.41E-04**	**18.01200**
**84**	**[M+H]+**	**390.20317**	**Propiverine**	**HMDB0041999**	**1.60836**	**4.38E-04**	**16.98400**
**85**	**[M+H]+**	**347.13970**	**Gibberellic acid**	**HMDB0003559**	**1.60810**	**4.51E-04**	**9.89540**
**86**	**[M+H]+**	**446.26972**	**Irbesartan**	**HMDB0015163**	**1.60807**	**4.59E-04**	**5.08650**
**87**	**[M+H]+**	**398.18019**	**(S)-Bitalin A 12-glucoside**	**HMDB0038328**	**1.60598**	**4.64E-04**	**113.43000**
**88**	**[M+H]+**	**473.15780**	**Geranylgeranyl pyrophosphate**	**HMDB0004486**	**1.60495**	**4.39E-04**	**20.55000**
**89**	**[M+H]+**	**455.17288**	**Methotrexate hydrate**	**HMDB0014703**	**1.60425**	**4.68E-04**	**24.21300**
**90**	**[M+H]+**	**472.15558**	**10-Formyldihydrofolate**	**HMDB0006485**	**1.60385**	**4.61E-04**	**34.30800**
**91**	**[M+H]+**	**471.15253**	**Armillaridin**	**HMDB0035134**	**1.60370**	**4.65E-04**	**31.49600**
**92**	**[M+H]+**	**791.43161**	**Melilotoside B**	**HMDB0041465**	**1.60263**	**4.92E-04**	**65.58200**

**93**	**[M+H]+**	**408.16791**	**3,4,5-trihydroxy-6-(2-hydroxy-1,2-diphenylethoxy)oxane-2-carboxylic acid**	**HMDB0135201**	**1.60176**	**5.03E-04**	**18.54000**
**94**	**[M+H]+**	**406.16961**	**Dihydrozeatin-O-glucoside**	**HMDB0012214**	**1.60024**	**4.92E-04**	**310.60000**
**95**	**[M+H]+**	**407.17233**	**Tadalafil**	**HMDB0014958**	**1.59933**	**5.05E-04**	**47.87800**
**96**	**[M+H]+**	**858.35660**	**Albanin H**	**HMDB0033754**	**1.59933**	**4.92E-04**	**259.88000**
**97**	**[M+H]+**	**417.16013**	**4R-Hydroxy solifenacin**	**HMDB0061127**	**1.59923**	**4.53E-04**	**4.98340**
**98**	**[M+H]+**	**841.38017**	**(3x,5x,10x)-9,10-Didehydroisohumbertiol O-[rhamnosyl-(1->4)-rhamnosyl-(1->2)-[rhamnosyl-(1->6)]-glucoside]**	**HMDB0040687**	**1.59917**	**5.30E-04**	**47.48600**
**99**	**[M+H]+**	**493.13611**	**3,4,5-trihydroxy-6-{[3-hydroxy-1-({4-methoxy-7-oxo-7H-furo[3,2-g]chromen-9-yl}oxy)-3-methylbutan-2-yl]oxy}oxane-2-carboxylic acid**	**HMDB0130148**	**1.59799**	**4.73E-04**	**27.65600**
**100**	**[M+H]+**	**404.22164**	**3-(2-{4-[2-(dimethylamino)ethoxy]phenyl}-3-ethyl-3-phenyloxiran-2-yl)phenol**	**HMDB0142485**	**1.59738**	**5.05E-04**	**71.64700**
**101**	**[M+H]+**	**956.27560**	**5-cis-8-cis-Tetradecadienoyl-CoA**	**HMDB0002288**	**1.59662**	**8.08E-04**	**2.18130**
**102**	**[M+H]+**	**278.11530**	**Isoetharine**	**HMDB0014366**	**1.59658**	**5.22E-04**	**19.04500**
**103**	**[M+H]+**	**812.35178**	**2-amino-4-({1-[(carboxymethyl)-C-hydroxycarbonimidoyl]-2-({4,10,11,14,16-pentahydroxy-5,9,11,13,13-pentamethyl-2-[1-(2-methyl-1,3-thiazol-4-yl)prop-1-en-2-yl]-12-oxo-1-azacyclohexadec-1(16)-en-5-yl}sulfanyl)ethyl}-C-hydroxycarbonimidoyl)butanoic acid**	**HMDB0127559**	**1.59651**	**5.53E-04**	**95.10500**
**104**	**[M+H]+**	**801.43386**	**Fumonisin FP3**	**HMDB0031828**	**1.59575**	**5.20E-04**	**65.34000**
**105**	**[M+H]+**	**346.13484**	**7-Hydroxy-6-methyl-8-ribityl lumazine**	**HMDB0004256**	**1.59326**	**5.48E-04**	**4.90430**
**106**	**[M+H]+**	**338.14724**	**6-oxo-famciclovir**	**HMDB0061100**	**1.59316**	**4.96E-04**	**24.56500**
**107**	**[M+H]+**	**286.10440**	**N4-Acetylcytidine**	**HMDB0005923**	**1.59289**	**4.79E-04**	**23.80000**
**108**	**[M+H]+**	**411.18385**	**Eremopetasitenin C3**	**HMDB0032772**	**1.59222**	**5.57E-04**	**31.10200**
**109**	**[M+H]+**	**412.18615**	**Dihydroxyfumitremorgin C**	**HMDB0038581**	**1.59212**	**5.37E-04**	**28.31400**
**110**	**[M+H]+**	**410.18136**	**Marmesin rhamnoside**	**HMDB0039564**	**1.59185**	**5.49E-04**	**32.88100**
**111**	**[M+H]+**	**409.17782**	**Beclomethasone**	**HMDB0014538**	**1.59178**	**5.44E-04**	**32.81800**
**112**	**[M+H]+**	**268.13280**	**Methionyl-Threonine**	**HMDB0028983**	**1.59154**	**5.21E-04**	**40.82400**
**113**	**[M+H]+**	**797.40119**	**Angiotensin IV**	**HMDB0001038**	**1.59153**	**5.29E-04**	**161.04000**
**114**	**[M+H]+**	**339.14878**	**Timolol**	**HMDB0014517**	**1.59075**	**4.87E-04**	**8.88290**

115	[M+H]+	393.19880	Betamethasone	HMDB0014586	1.59043	5.40E-04	6.84580
116	[M+H]+	389.20065	1-(4-Hydroxy-3,5-dimethoxyphenyl)-7-(4-hydroxy-3-methoxyphenyl)-3,5-heptanediol	HMDB0041091	1.58924	5.68E-04	59.67400
117	[M+H]+	618.24371	Taurocholic acid 3-sulfate	HMDB0002581	1.58887	6.07E-04	9.84440
118	[M+H]+	388.19783	Terazosin	HMDB0015293	1.58818	5.73E-04	84.20600
119	[M+H]+	218.08329	Captopril	HMDB0015328	1.58817	5.56E-04	12.43800
120	[M+H]+	387.19441	(4E)-6-hydroxy-1-(4-hydroxy-3-methoxyphenyl)tetradec-4-en-3-one	HMDB0137416	1.58733	5.80E-04	83.79200
121	[M+H]+	105.06807	Cyclohexene	-	1.58730	5.81E-04	46.98400
122	[M+H]+	263.09765	Asp-phe	HMDB0000706	1.58689	5.73E-04	18.19100
123	[M+H]+	428.15561	Lucuminamide	HMDB0031697	1.58676	6.46E-04	17.59600
124	[M+H]+	425.15297	Eprosartan	HMDB0015014	1.58603	6.24E-04	15.19100
125	[M+H]+	329.25743	Stanozolol	HMDB0003116	1.58583	8.56E-04	13.89200
126	[M+H]+	121.06372	4-Vinylphenol	HMDB0004072	1.58545	5.92E-04	12.22500
127	[M+H]+	757.47127	Lycoperoside D	HMDB0034484	1.58541	6.33E-04	16.69300
128	[M+H]+	265.11336	N-γ-Acetyl-N-2-Formyl-5-methoxykynurenamine	HMDB0004259	1.58541	5.95E-04	22.60800
129	[M+H]+	370.18619	Coriandrone D	HMDB0029972	1.58511	5.86E-04	41.20300
130	[M+H]+	213.07136	Hexanethioic acid S-propyl ester	HMDB0039466	1.58493	5.82E-04	11.65600
131	[M+H]+	427.15419	Chitobiose	HMDB0003556	1.58487	6.45E-04	15.84300
132	[M+H]+	369.18206	11-hydroxy-Delta(9)-tetrahydrocannabinol	HMDB0062581	1.58461	6.10E-04	40.65300
133	[M+H]+	145.04989	2-Methylcinnamic acid	-	1.58442	6.45E-04	13.98300
134	[M+H]+	153.03909	Oxypurinol	HMDB0000786	1.58396	5.79E-04	11.84700
135	[M+H]+	401.16032	3-[3,4-dihydroxy-5-(3-methylbut-2-en-1-yl)phenyl]-1-(2,4-dihydroxyphenyl)propan-1-one	HMDB0135886	1.58354	6.02E-04	4.26350
136	[M+H]+	422.15001	Reduced haloperidol	HMDB0060903	1.58297	6.45E-04	13.69700
137	[M+H]+	426.15653	2-{9-hydroxy-2-oxo-2H,8H,9H-furo[2,3-h]chromen-8-yl}propan-2-yl (2E)-3-(4-hydroxyphenyl)prop-2-enoate	HMDB0128937	1.58286	6.60E-04	14.59200
138	[M+H]+	609.28367	Reserpine	HMDB0014351	1.58148	5.81E-04	8.45330
139	[M+H]+	481.17885	Flupenthixol	HMDB0015013	1.58141	6.15E-04	15.82700

140	[M+H]+	591.27979	Protoporphyrin IX dimethyl ester	HMDB0000810	1.57961	6.59E-04	190.96000
141	[M+H]+	431.17673	Geranylgeranyl-PP	HMDB0004486	1.57940	6.57E-04	102.12000
142	[M+H]+	414.17133	Flavoxate	HMDB0015279	1.57936	8.03E-04	7.51770
143	[M+H]+	385.17818	Corticosterone	HMDB0001547	1.57869	6.52E-04	14.60100
144	[M+H]+	608.29441	Leukotriene C4	HMDB0001198	1.57868	6.10E-04	5.41100
145	[M+H]+	106.07143	2-Amino-2-methyl-1,3-propanediol	-	1.57777	6.30E-04	26.93300
146	[M+H]+	215.06717	3-Phenoxybenzoic acid	HMDB0041807	1.57767	6.35E-04	10.29900
147	[M+H]+	600.27249	Gluten exorphin A5	HMDB0059793	1.57610	6.85E-04	261.29000
148	[M+H]+	417.15727	6-Gingesulfonic acid	HMDB0038999	1.57590	6.59E-04	8.98680
149	[M+H]+	418.16760	Capecitabine	HMDB0015233	1.57583	7.11E-04	110.27000
150	[M+H]+	433.25827	Hydrocortisone 17-butyrate	HMDB0000063	1.57573	6.54E-04	12.55500
151	[M+H]+	586.41839	33-Deoxy-33-hydroperoxyfurohyperforin	HMDB0038387	1.57486	8.69E-04	0.24064
152	[M+H]+	447.16903	Cetirizine	HMDB0005032	1.57448	6.95E-04	9.51970
153	[M+H]+	282.11244	6-Methylaminopurine arabinoside	-	1.57420	6.94E-04	10.00500
154	[M+H]+	433.18043	Cyproterone	HMDB0015587	1.57406	7.01E-04	8.75880
155	[M+H]+	448.17295	Glisoxepide	HMDB0015406	1.57404	7.09E-04	8.86820
156	[M+H]+	463.16378	PSF-A	HMDB0034608	1.57322	6.45E-04	6.06230
157	[M+H]+	813.35472	5-{[2,2-dimethyl-7-(2-methylbut-3-en-2-yl)-5-[(2-methylbut-3-en-2-yl)oxy]-8-oxo-2H,8H-pyrano[3,2-g]chromen-10-yl]oxy}-8,8-dimethyl-10-(2-methylbut-3-en-2-yl)-3-[2-(oxiran-2-yl)propan-2-yl]-2H,8H-pyrano[3,2-g]chromen-2-one	HMDB0133018	1.57281	6.82E-04	44.04600
158	[M+H]+	766.48125	PE(20:5(5Z,8Z,11Z,14Z,17Z)/20:5(5Z,8Z,11Z,14Z,17Z))	HMDB0009467	1.57195	9.29E-04	6.41250
159	[M+H]+	601.27111	APC	HMDB0060661	1.57185	7.27E-04	58.97200
160	[M+H]+	406.23851	LysoPE(0:0/14:0)	HMDB0011470	1.57043	9.39E-04	0.24022
161	[M+H]+	482.18271	Adefovir Dipivoxil	HMDB0014856	1.57039	7.07E-04	15.45400
162	[M+H]+	802.42830	14-hydroxyclarithromycin	HMDB0061019	1.57029	6.67E-04	30.15100
163	[M+H]+	592.29393	<i>ortho</i>-hydroxyatorvastatin	HMDB0061015	1.57027	7.66E-04	15.97400

164	[M+H]+	859.35977	Licoricesaponin E2	HMDB0038719	1.56981	6.57E-04	55.41000
165	[M+H]+	249.11777	Pentobarbital	HMDB0014457	1.56913	7.65E-04	13.23400
166	[M+H]+	129.05392	Dihydro-4,4-dimethyl-2,3-furandione	-	1.56670	7.32E-04	10.09200
167	[M+H]+	543.08883	Melitric acid B	HMDB0040680	1.56210	6.78E-04	31.54700
168	[M+H]+	419.17031	8-Propanoylneosolaniol	HMDB0038562	1.56123	8.43E-04	12.07500
169	[M+H]+	127.03762	2,3-Dimethylmaleicanhydride	HMDB0003243	1.56107	8.56E-04	7.96780
170	[M+H]+	598.38853	LysoPC(22:2(13Z,16Z))	HMDB0010400	1.56059	7.06E-04	8.83160
171	[M+H]+	214.07423	Debrisoquine	HMDB0006543	1.55918	7.01E-04	12.15100
172	[M+H]+	506.17646	3-cis-Hydroxyglipizide	HMDB0060934	1.55914	7.80E-04	19.66300
173	[M+H]+	770.16967	FADH	HMDB0001197	1.55406	9.56E-04	7.46240
174	[M+H]+	434.18139	3-(3,4-dimethoxyphenyl)-N-[2-(3,4-dimethoxyphenyl)ethyl]-3-hydroxypropanimidic acid	HMDB0135477	1.55059	9.52E-04	3.80970
175	[M+H]+	564.37821	Hovenidulcigenin B	HMDB0041547	1.54663	8.65E-04	5.74750
176	[M+H]+	416.15312	Trazodone	HMDB0014794	1.54558	8.73E-04	7.56050
177	[M+H]+	399.26048	10';-Apo-beta-caroten-10';-al	HMDB0036887	1.54451	1.13E-03	0.30370
178	[M+H]+	106.05644	L-Serine	HMDB0000187	1.54373	8.51E-04	0.46695
179	[M+H]+	342.17059	3-(3,4-dimethoxyphenyl)-N-[2-(4-hydroxy-3-methoxyphenyl)ethyl]propanimidic acid	HMDB0135481	1.54320	9.52E-04	5.40960
180	[M+H]+	693.31575	Allodesmosine	HMDB0040704	1.53982	1.43E-03	16.96900
181	[M+H]+	489.24780	Prednicarbate	HMDB0015262	1.53697	1.09E-03	58.40500
182	[M+H]+	374.24719	Undecanoylcarnitine	HMDB0013321	1.53628	1.35E-03	0.23171
183	[M+H]+	392.27027	Tsangane L 3-glucoside	HMDB0040824	1.53366	1.52E-03	0.19207
184	[M+H]+	428.17364	7-Hydroxydehydroglaucine	HMDB0033084	1.53328	1.14E-03	6.94940
185	[M+H]+	414.25360	Isopetasoside	HMDB0029622	1.53267	1.49E-03	0.19679
186	[M+H]+	375.25044	2-(14,15-Epoxyeicosatrienoyl) Glycerol	HMDB0013651	1.52867	1.46E-03	0.23653
187	[M+H]+	420.25559	PC-M6	HMDB0038568	1.52840	1.45E-03	0.23576
188	[M+H]+	421.25875	9';-Carboxy-gamma-chromanol	HMDB0012868	1.52781	1.46E-03	0.23717
189	[M+H]+	445.16025	Mesoridazine	HMDB0015068	1.52004	1.33E-03	5.29970

**190**	**[M+H]+**	**420.17245**	**2-[4-(4-chloro-1,2-diphenylbut-1-en-1-yl)phenoxy]-1-(dimethylamino)ethan-1-ol**	**HMDB0143643**	**1.51803**	**1.40E-03**	**5.93090**
**191**	**[M+H]+**	**461.23957**	**DHAP(18:0e)**	**HMDB0011142**	**1.51599**	**1.57E-03**	**0.42882**
**192**	**[M+H]+**	**390.24246**	**3-hydroxyundecanoyl carnitine**	**HMDB0061637**	**1.51351**	**1.74E-03**	**0.23506**
**193**	**[M+H]+**	**376.25313**	**PGD2 ethanolamide**	**HMDB0013629**	**1.51082**	**1.78E-03**	**0.23669**
**194**	**[M+H]+**	**732.22539**	**Scleroglucan**	**HMDB0029948**	**1.50974**	**1.76E-03**	**36.00800**
**195**	**[M+H]+**	**233.10796**	**Methaqualone**	**HMDB0240285**	**1.50553**	**1.34E-03**	**3.66540**
**196**	**[M+H]+**	**398.25737**	**[6]-Gingerdiol 3,5-diacetate**	**HMDB0040568**	**1.50428**	**1.75E-03**	**0.26007**
**197**	**[M+H]+**	**321.28802**	**Cis-8,11,14-Eicosatrienoic acid Methyl ester**	**-**	**1.49885**	**2.06E-03**	**3.06220**
**198**	**[M+H]+**	**388.22698**	**Dibucaine**	**HMDB0014668**	**1.49116**	**2.16E-03**	**0.24792**
**199**	**[M+H]+**	**422.26134**	**N-Palmitoyltaurine**	**HMDB0240594**	**1.49006**	**2.14E-03**	**0.24590**
**200**	**[M+H]+**	**391.24518**	**Methyl-[10]-shogaol**	**HMDB0031465**	**1.48755**	**2.26E-03**	**0.28206**
**201**	**[M+H]+**	**389.23347**	**Digoxigenin**	**HMDB0060731**	**1.48702**	**2.26E-03**	**0.27134**
**202**	**[M+H]+**	**896.61165**	**PE(22:6(4Z,7Z,10Z,13Z,16Z,19Z)/24:1(15Z))**	**HMDB0009707**	**1.48094**	**2.74E-03**	**2.80060**
**203**	**[M+H]+**	**404.22317**	**LysoPE(14:1(9Z)/0:0)**	**HMDB0011501**	**1.47426**	**2.57E-03**	**0.28813**
**204**	**[M+H]+**	**392.07647**	**6';';-O-Acetylholocalin**	**HMDB0036331**	**1.47175**	**2.55E-03**	**0.34640**
**205**	**[M+H]+**	**827.71718**	**TG(14:0/18:3(6Z,9Z,12Z)/18:1(11Z))**	**HMDB0042550**	**1.46496**	**3.23E-03**	**2.54370**
**206**	**[M+H]+**	**201.10784**	**Sebacate**	**HMDB0000792**	**1.46309**	**3.13E-03**	**0.43063**
**207**	**[M+H]+**	**587.42263**	**Ginsenoside Rh3**	**HMDB0039645**	**1.46031**	**3.00E-03**	**0.28778**
**208**	**[M+H]+**	**423.26181**	**Quinestrol**	**HMDB0015579**	**1.45602**	**3.12E-03**	**0.37670**
**209**	**[M+H]+**	**370.27086**	**Anandamide**	**HMDB0004080**	**1.45557**	**2.63E-03**	**0.49334**
**210**	**[M+H]+**	**860.77708**	**TG(15:0/16:0/20:3(5Z,8Z,11Z))**	**HMDB0043029**	**1.45053**	**3.47E-03**	**2.96560**
**211**	**[M+H]+**	**396.99989**	**[9-hydroxy-2-(2-hydroxypropan-2-yl)-7-oxo-2H,3H,7H-furo[3,2-g]chromen-3-yl]oxidanesulfonic acid**	**HMDB0132925**	**1.44938**	**3.00E-03**	**0.27775**
**212**	**[M+H]+**	**393.30138**	**Deoxycholic acid**	**HMDB0000626**	**1.44819**	**3.12E-03**	**0.27404**
**213**	**[M+H]+**	**414.23231**	**1-Hexanol arabinosylglucoside**	**HMDB0031689**	**1.44231**	**3.19E-03**	**0.29117**
**214**	**[M+H]+**	**717.25530**	**Hv-NCC-1**	**HMDB0039005**	**1.43825**	**3.72E-03**	**46.47200**
**215**	**[M+H]+**	**316.16592**	**Isoleucyl-Tryptophan**	**HMDB0028918**	**1.43432**	**3.61E-03**	**0.26966**

216	[M+H]+	394.30454	10';-Apo-beta-carotenal	HMDB0059605	1.43284	3.54E-03	0.28203
217	[M+H]+	310.96999	alpha-Trichloromethylbenzyl acetate	HMDB0061807	1.42973	7.75E-03	0.44621
218	[M-H]-	135.02277	Hypoxanthine	HMDB0000157	1.42189	6.83E-03	0.48385
219	[M-H]-	417.25613	Pangamic acid	HMDB0029949	1.41408	4.64E-03	0.36366
220	[M-H]-	405.22880	Annoglabasin C	HMDB0036263	1.41403	4.23E-03	0.38101
221	[M-H]-	379.28226	MG(18:1(9Z)/0:0/0:0)	HMDB0011567	1.39720	5.10E-03	0.32689
222	[M-H]-	580.31001	19-Hydroxycinnzeylanol 19-glucoside	HMDB0036856	1.39575	3.75E-03	2.24500
223	[M-H]-	419.24608	Calycanthidine	HMDB0030281	1.37714	5.81E-03	0.38727
224	[M-H]-	599.42297	Schottenol 3-glucoside	HMDB0034185	1.37554	5.95E-03	0.35492
225	[M-H]-	186.22060	Dodecylamine	-	1.37332	7.75E-03	0.36725
226	[M-H]-	600.15133	5';-Methoxybilobetin	HMDB0038101	1.36895	6.95E-03	2.16030
227	[M-H]-	377.25814	Floionolic acid	HMDB0034295	1.35749	7.45E-03	0.27578
228	[M-H]-	378.27978	Sphingosine 1-phosphate (d19:1-P)	HMDB0060062	1.34891	7.49E-03	0.27999
229	[M-H]-	775.54494	PG(18:0/18:2(9Z,12Z))	HMDB0010605	1.34233	7.87E-03	0.29707
230	[M-H]-	376.27268	Kinetensin 1-3	HMDB0012983	1.34059	8.04E-03	0.27602
231	[M-H]-	377.27681	5,7-Docosanedione	HMDB0035568	1.33883	8.14E-03	0.27854
232	[M-H]-	773.53825	PG(18:2(9Z,12Z)/18:1(11Z))	HMDB0010648	1.33419	8.28E-03	0.28222
233	[M-H]-	251.20342	Sclareolide	HMDB0035293	1.32698	1.23E-02	2.01890
234	[M-H]-	458.27168	Tirofiban	HMDB0014913	1.31711	1.05E-02	0.49149
235	[M-H]-	774.54198	PE(P-16:0/22:4(7Z,10Z,13Z,16Z))	HMDB0011358	1.31296	9.71E-03	0.29196
236	[M-H]-	508.06128	N(6)-(1,2-dicarboxyethyl)AMP	HMDB0059653	1.29583	1.20E-02	0.45344
237	[M-H]-	395.24972	(3R, 6';Z)-3,4-Dihydro-8-hydroxy-3-(6-pentadecenyl)-1H-2-benzopyran-1-one	HMDB0041301	1.29533	1.19E-02	0.28686
238	[M-H]-	317.16841	Methylscopolamine	HMDB0014605	1.29497	1.10E-02	0.48020
239	[M-H]-	375.08596	Neodiospyrin	HMDB0029538	1.28998	1.16E-02	0.33971

Table S2. List of the differentiated metabolites in lung tissue.

NO.	Ion Form	m/z(Da)	Compound name	HMDB	VIP	P value	Fold change
1	[M+H]+	271.23079	MG(0:0/a-13:0/0:0)[rac]	HMDB0072843	1.44520	1.73E-04	260.71000
2	[M+H]+	248.14258	Lansiumamide C	HMDB0038838	1.68087	6.28E-04	0.29155
3	[M+H]+	624.30540	Leukotriene C4	HMDB0001198	1.71768	6.90E-04	2.79150
4	[M+H]+	552.37467	Pentacosanoylglycine	HMDB0013297	1.16654	1.38E-02	0.41707
5	[M+H]+	439.07178	3-O-alpha-D-Glucopyranuronosyl-D-xylose	HMDB0039723	1.27650	3.65E-03	2.18930
6	[M+H]+	241.11777	γ-Glu-Leu	HMDB0011171	1.28916	3.06E-03	2.09230
7	[M+H]+	309.10669	L-alpha-Amino-1H-pyrrole-1-hexanoic acid	HMDB0040551	1.24455	5.39E-03	2.08460
8	[M+H]+	645.32497	3-O-Protocatechuoylceanothic acid	HMDB0029625	1.79770	2.10E-04	2.00080
9	[M+H]+	365.34539	Nervonic acid	HMDB0002368	1.57812	9.25E-09	0.02464
10	[M+H]+	319.26282	Methyl arachidonate	HMDB0062594	1.55731	1.29E-06	0.04615
11	[M+H]+	338.34151	Erucamide	HMDB0244507	1.54925	9.26E-07	0.05274
12	[M+H]+	295.26344	9,12-Octadecadienoic acid (Z,Z)-, methyl ester	HMDB0034381	1.41781	3.76E-04	0.09822
13	[M+H]+	293.28255	Octadecanol	HMDB0002350	1.45789	1.12E-04	0.10975
14	[M+H]+	311.29698	Eicosanoic acid	HMDB0002212	1.57467	1.17E-08	0.11599
15	[M+H]+	599.32889	Ciclesonide	HMDB0015480	1.55998	4.86E-07	0.12871
16	[M+H]+	836.60938	PC(24:1(15Z)/14:1(9Z))	HMDB0008789	1.57244	1.93E-07	0.13232
17	[M+H]+	835.60489	PE-NMe2(18:3(6Z,9Z,12Z)/22:4(7Z,10Z,13Z,16Z))	HMDB0114117	1.57856	2.33E-08	0.13858
18	[M+H]+	378.20881	4-(dimethylamino)-1-(3-hydroxyphenyl)-3-methyl-2-phenylbutan-2-yl propanoate	HMDB0144284	1.54728	3.21E-06	0.14805
19	[M+H]+	600.33282	LysoPC(20:5(5Z,8Z,11Z,14Z,17Z))	HMDB0010397	1.54158	4.58E-06	0.15169
20	[M+H]+	320.26540	Retinyl ester	HMDB0003598	1.49694	4.00E-05	0.15381
21	[M+H]+	321.31138	Arachidyl alcohol	HMDB0011619	1.39209	8.25E-04	0.17870
22	[M+H]+	834.60088	PE-NMe(22:6(4Z,7Z,10Z,13Z,16Z,19Z)/20:0)	HMDB0113685	1.58240	1.73E-09	0.20308
23	[M+H]+	788.52421	PE(20:4(5Z,8Z,11Z,14Z)/20:4(8Z,11Z,14Z,17Z))	HMDB0009400	1.55700	5.66E-07	4.83870
24	[M+H]+	378.26374	(4Z,7Z,11E,13Z,16Z,19Z)-10-Hydroperoxydocosahexaenoic acid	HMDB0062277	1.15539	1.47E-02	0.20735

**25**	**[M+H]+**	**507.36229**	**Isomultiflorenyl acetate**	**HMDB0038062**	**1.50730**	**1.36E-05**	**0.21284**
**26**	**[M+H]+**	**284.29306**	**Stearamide**	**HMDB0034146**	**1.48498**	**1.04E-04**	**4.66330**
**27**	**[M+H]+**	**335.29267**	**2,2,6,10,14-Pentamethylpentadecanoic acid**	**HMDB0031318**	**1.36524**	**1.74E-03**	**0.21811**
**28**	**[M+H]+**	**297.28037**	**Methyl stearate**	**HMDB0034154**	**1.56529**	**5.09E-07**	**0.23019**
**29**	**[M+H]+**	**297.27566**	**Cis-10-Nonadecenoic acid**	**HMDB0013622**	**1.50257**	**3.20E-05**	**0.27095**
**30**	**[M+H]+**	**414.15466**	**6,8-Di-O-methylaverufin**	**HMDB0035465**	**1.13392**	**1.73E-02**	**3.59220**
**31**	**[M+H]+**	**764.52444**	**PE-NMe2(22:5(4Z,7Z,10Z,13Z,16Z)/14:1(9Z))**	**HMDB0114524**	**1.29265**	**4.33E-03**	**3.44030**
**32**	**[M+H]+**	**831.57491**	**PE-NMe2(18:3(6Z,9Z,12Z)/22:6(4Z,7Z,10Z,13Z,16Z,19Z))**	**HMDB0114120**	**1.30405**	**3.42E-03**	**3.43230**
**33**	**[M-H]-**	**765.52752**	**Narasin**	**HMDB0030448**	**1.29486**	**4.27E-03**	**3.36550**
**34**	**[M-H]-**	**386.35381**	**Fistulosin**	**HMDB0033637**	**1.25911**	**7.31E-03**	**3.34290**
**35**	**[M-H]-**	**256.26298**	**Hexadecanamide**	**HMDB0012273**	**1.19274**	**1.43E-02**	**3.32690**
**36**	**[M-H]-**	**830.57111**	**PE-NMe(20:3(5Z,8Z,11Z)/22:5(4Z,7Z,10Z,13Z,16Z))**	**HMDB0113390**	**1.30956**	**3.16E-03**	**3.19830**
**37**	**[M-H]-**	**613.47489**	**DG(18:3(9Z,12Z,15Z)/16:0/0:0)**	**HMDB0007301**	**1.32088**	**3.42E-03**	**0.32696**
**38**	**[M-H]-**	**339.20167**	**Piperochromenoic acid**	**HMDB0040635**	**1.19305**	**1.21E-02**	**2.96380**
**39**	**[M-H]-**	**331.26268**	**All-cis-4,7,10,13,16-docosapentaenoic acid**	**HMDB0001976**	**1.37496**	**1.61E-03**	**0.34610**
**40**	**[M-H]-**	**547.29268**	**2,3-Dihydrowithanolide E**	**HMDB0034057**	**1.20453**	**1.38E-02**	**2.88800**
**41**	**[M-H]-**	**837.59981**	**DG(13D5/13M5/0:0)**	**HMDB0116410**	**1.39820**	**8.83E-04**	**0.35741**
**42**	**[M-H]-**	**703.57676**	**SM(d18:0/16:1(9Z))**	**HMDB0013464**	**1.38537**	**1.20E-03**	**0.38511**
**43**	**[M-H]-**	**368.34289**	**Oleoylcholine**	**HMDB0240596**	**1.29592**	**5.90E-03**	**2.55400**
**44**	**[M-H]-**	**442.14288**	**Dihydrofolic acid**	**HMDB0001056**	**1.15504**	**1.39E-02**	**2.47990**
**45**	**[M-H]-**	**484.27495**	**LysoPE(14:0/0:0)**	**HMDB0011500**	**1.33835**	**3.20E-03**	**0.40827**
**46**	**[M-H]-**	**339.33895**	**Erucic acid**	**HMDB0002068**	**1.28799**	**4.04E-03**	**0.42859**
**47**	**[M-H]-**	**527.36221**	**Dilauryl 3,3';-thiodipropionate**	**HMDB0038474**	**1.41060**	**5.13E-04**	**2.20260**
**48**	**[M-H]-**	**705.58496**	**DG(22:2n6/0:0/22:5n3)**	**HMDB0056349**	**1.32940**	**3.11E-03**	**0.45939**
**49**	**[M-H]-**	**389.22442**	**Arginyl-Arginine**	**HMDB0028703**	**1.19774**	**1.07E-02**	**2.15140**
**50**	**[M-H]-**	**360.18381**	**Enrofloxacin**	**HMDB0029861**	**1.37669**	**1.49E-03**	**0.46953**

**51**	**[M-H]-**	**238.02957**	**2-(4-Methyl-5-thiazolyl)ethyl propionate**	**HMDB0032424**	**1.27994**	**6.88E-03**	**2.09980**
**52**	**[M-H]-**	**896.54198**	**PGP(20:4(5Z,8Z,11Z,14Z)/18:0)**	**HMDB0116482**	**1.34215**	**2.79E-03**	**0.47648**
**53**	**[M-H]-**	**157.06151**	**L-β-Imidazolelactic acid**	**HMDB0002320**	**1.34753**	**1.29E-03**	**0.47753**
**54**	**[M-H]-**	**526.35795**	**Daphniphylline**	**HMDB0030291**	**1.42284**	**4.21E-04**	**2.06410**
**55**	**[M-H]-**	**334.18428**	**Glucosyl (E)-2,6-Dimethyl-2,5-heptadienoate**	**HMDB0035136**	**1.56187**	**3.22E-07**	**0.48606**
**56**	**[M-H]-**	**265.14855**	**Isoleptospermone**	**HMDB0041425**	**1.53296**	**7.83E-06**	**2.02950**
